# First evidence of maternally inherited mosaicism in *TGFBR1* and subtle primary myocardial changes in Loeys-Dietz syndrome: a case report

**DOI:** 10.1186/s12881-018-0661-2

**Published:** 2018-09-15

**Authors:** Anwar Baban, Monia Magliozzi, Bart Loeys, Rachele Adorisio, Viola Alesi, Aurelio Secinaro, Bernadette Corica, Luca Vricella, Harry C. Dietz, Fabrizio Drago, Antonio Novelli, Antonio Amodeo

**Affiliations:** 1 Pediatric Cardiology and Cardiac Arrhythmia/Syncope Unit, Department of Pediatric Cardiology and Cardiac Surgery, Bambino Gesù Children’s Hospital and Research Institute, Piazza S. Onofrio, 4, 00165 Rome, Italy; 2 Laboratory of Medical Genetics, Bambino Gesù Children’s Hospital and Research Institute, Rome, Italy; 3 Center of Medical Genetics, Faculty of Medicine and Health Sciences, University of Antwerp and Antwerp University Hospital, Antwerp, Belgium; 4 Department of Imaging, Bambino Gesù Children’s Hospital and Research Institute, IRCCS, Rome, Italy; 5 Division of Cardiothoracic Surgery, Department of Surgery, the Johns Hopkins University School of Medicine, Baltimore, MD USA; 6 Department of Medicine, The McKusick-Nathans Institute of Genetic Medicine, The Johns Hopkins Medical Institutions, Baltimore, MD USA; 7 Mechanical Assistance Device and Artificial Heart Unit, Department of Pediatric Cardiology and Cardiac Surgery, Bambino Gesù Children’s Hospital and Research Institute, Rome, Italy

**Keywords:** Loeys-Dietz syndrome (LDS), Oculo cutaneous albinism (OCA), Mosaicism, *TGFBR1*, *TGFBR2*, Aortic dilatation

## Abstract

**Background:**

Loeys-Dietz syndrome (LDS) is a rare multisystemic disorder characterized by vascular and skeletal abnormalities, with considerable intra- and interfamilial variability.

**Case presentation:**

We report the case of an 8-year-old male with clinical features of two distinct genetic disorders, namely LDS, manifesting in the first months by progressive aortic root dilatation, arterial tortuosity, bifid uvula, and inguinal hernias and oculocutaneous albinism (OCA) manifesting by white hair and skin that does not tan, nystagmus, reduced iris pigment with iris translucency, and reduced retinal pigment). We identified previously reported, homozygous mutations of *TYR*, c.1A > G (p.Met1Val) and heterozygous, missense mutation of *TGFBR1*, c.1460G > A (p.Arg487Gln). Family history revealed that his mother underwent multiple surgical repairs for recurrent hemorrhage originating from the buccal artery. Molecular studies confirmed a maternally inherited low grade *TGFBR1* mutation somatic mosaicism (18% in peripheral blood leukocytes, 18% in buccal cells and 10% in hair root cells). Maternal cardiac investigations revealed peculiar cardiovascular features: mild tortuosity at the aortic arch, dilatation of the proximal abdominal aorta, multiple deep left ventricular myocardial crypts, and dysplastic mitral valve. *TGFBR2* germline mosaicism has been described in three fathers of children carrying *TGFBR2* mutations but, to the best of our knowledge, no case of maternally inherited *TGFBR1* mutation mosaicism has been reported so far.

**Conclusions:**

This case report suggests that individuals with somatic mosaicism might be at risk for mild and unusual forms of LDS but germline mosaicism can lead to full blown picture of the disease in offspring.

**Electronic supplementary material:**

The online version of this article (10.1186/s12881-018-0661-2) contains supplementary material, which is available to authorized users.

## Background

Loeys-Dietz syndrome (LDS) is a rare genetic disorder characterized by progressive vascular manifestations (cerebral, thoracic, and abdominal arterial/aortic aneurysms and/or dissections) and skeletal abnormalities (sternal anomalies, scoliosis, joint laxity, arachnodactyly, talipes equinovarus). LDS is classified according to the underlying causative gene mutation in type 1 (*TGFBR1*), type 2 (*TGFBR2*), type 3 (*SMAD3*), type 4 (*TGFB2*), type 5 (*TGFB3*), and type 6 (*SMAD2*)*.* The detection rate for causative mutations is high. No differences in phenotype are observed between individuals with mutations in *TGFBR1* and *TGFBR2* [1]. Somatic mosaicism is a rare event in these conditions and specific phenotype-genotype correlations are not yet documented. In addition, myocardial involvement and dysfunction as well as the potential embryologic correlation between transforming growth factor receptor mutations and myocardial changes have rarely been described in the literature. In this report, a literature review on these topics in LDS is also included.

## Case presentation

The index patient is the only male child of Caucasian Italian non-consanguineous parents. He was born at term. Maternal and paternal age at birth was 42 and 50 years, respectively. There was no prenatal exposure to maternal smoking, alcohol no medications. Second trimester ultrasound scan showed mild aortic root dilatation. Birth weight and length were 4.5 kg (98th percentile) and 54 cm (99th percentile), respectively. He reached all milestones at normal ages (walking and first words by 13–14 months). Normal schooling was reported.

At birth, aortic dilatation was confirmed. Cardiac follow-up was accomplished at another institution where the diagnosis of OCA was also established based on cutaneous features (white hair and white skin that does not tan) and ocular findings (nystagmus, reduced iris pigment with iris translucency, and reduced retinal pigment with visualization of choroidal blood vessels on ophthalmoscopic examination). Surgical correction of bilateral inguinal hernias was also undertaken in early childhood.

Progressive aortic root and ascending aorta dilatation was monitored echocardiographically each year. No medication was prescribed. The child had progressive worsening in functional class Ross NYHA from I to II-III prior to coming to our attention. He never experienced acute events (syncope nor palpitations).

The child came to our attention at the age of 7 years and 4 months. Growth parameters revealed macrosomia with weight 31.5 kg, height 138 cm and OFC 55 cm (97th, 99th and 75th–95th percentile, respectively). Physical examination showed OCA, nystagmus, bifid uvula, joint laxity, bilateral pes planus, and no dysmorphic facial features. Echocardiography revealed severe dilatation of the aortic root and ascending aorta with effacement of the sino-tubular junction. Vascular magnetic resonance angiography (MRA) from head to pelvis showed diffuse vascular tortuosity of the intracranial vessels without evidence of aneurysm or dissection. A large left cererbal arachnoid cystic formation in the frontal insular region (diameters 87 × 67 × 32 mm) was observed, with significant pressure effect on the surrounding cortex and mild right-sided hemispheric shift from the midline. MRA confirmed that the aorta was the most affected segment of the vascular tree, with severe dilatation of the aortic root (39 × 38 mm - z score + 5.84) and ascending aorta (47 × 48 mm - z score + 10.18) with complete loss of the sino-tubular junction. The right coronary artery, the distal aortic arch and isthmus were severely angulated. However, the aortic arch was not excessively dilated (23 mm - z score + 2.35) whereas the descending aorta was mildly dilated (20 × 20 mm - z score + 2.91). Abdominal vascular imaging was normal except for marked tortuosity without significant dilatation of the distal branches of the splenic and gastroduodenal arteries.

Both parents had normal echocardiographic findings at baseline visit. The mother reported a history of 3 surgeries at the age of 48 years for recurrent hemorrhage originating from the right buccal artery. Recently, she also experienced severe reduction in visual acuity of her right eye due to spontaneous vitreous detachment. On physical examination, no craniofacial abnormalities were observed, except for a pinched nasal bridge.

The clinical features of the proband led to the diagnosis of two distinct genetic defects: OCA and LDS. A literature review (PubMed) and the investigation of specific dysmorphology search tools (London Medical Databases) confirmed the lack of prior reporting of the association of albinism with LDS. In order to exclude a contiguous gene syndrome, Chromosomal Microarray Analysis (CMA) at 100Kb resolution was performed using Agilent 4x180K oligo array platform (Agilent technologies, Santa Clara, CA, USA) according to the manufacturer’s protocol. Array scanning data were generated on the Agilent DNA Microarray Scanner and the results were analyzed by Cytogenomics software.

**Fig. 1:**
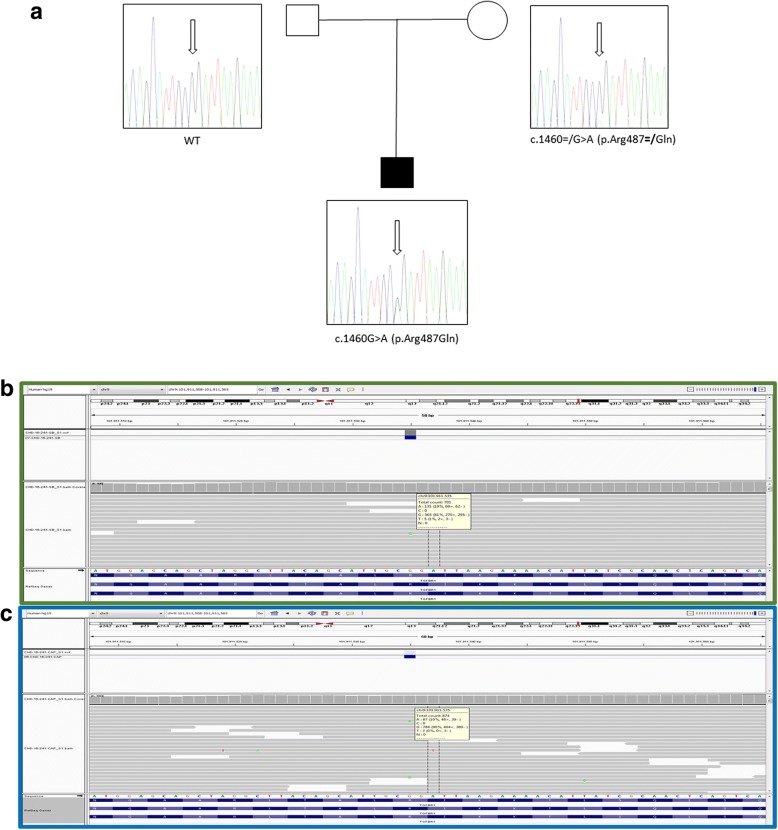
Family trios Sequence analysis on genomic DNA extracted from peripheral blood shows the heterozygous missense mutation c.1460G > A (p.Arg487Gln) in *TGFBR1*. Genetic characterization shows proband heterozygous status while the mother is in mosaic heterozygous (**a**). Mosaic*TGFBR1* mutation in maternal samples. Next-generation sequencing detects a heterozygous c.1460G > A (p.Arg487Gln) mutation in 18% in buccal cells (**b**) and 10% in hair root cells (**c**)

CMA turned out to be negative for copy number variation. A next generation sequencing (NGS) approach for molecular genetic study of the patient was performed through aortopathy NGS gene panel. Specifically, the following genes associated with aortopathy were analyzed: *ACTA2, COL3A1, FBN1, FBN2, MYH11, MYLK, SLC2A10, SMAD3, TGFB2, TGFBR1, TGFBR2, ELN, FLNA,* and *SMAD6*, by targeted resequencing, using a uniquely customized design (Nextera Rapid Capture Custom Enrichment Kit; Illumina, San Diego, CA), and sequenced with the MiSeq® sequencing platform (Illumina, San Diego, CA). A previously reported heterozygous, missense mutation in *TGFBR1* (c.1460G > A, p.Arg487Gln) [2] was identified This finding was validated by Sanger sequencing using standard protocols, and the DNA of the patient’s parents was tested for segregation studies, which identified a maternally inherited mutation in somatic mosaicism. Specifically, the rate of mosaicism was 18% in peripheral blood leukocytes, 18% in buccal cells and 10% in hair root cells (Fig. 1a, b). Sequencing of *OCA3* (*TYRP1*), mapping on the same chromosome as *TGFBR1*, was negative. Moreover, molecular analysis of TYR confirmed a previously described homozygous mutation of c.1A > G (p.Met1Val). Homozygous or compound heterozygous inactivating mutations in *TYR* lead to OCA type A, a recessive condition perfectly explaining in our patient the clinical features related to this specific disorder. As expected, trios analysis of the mutation showed both parents in heterozygous carrier status.

The child was operated for aortic root replacement complicated by complete atrioventricular block that required pacemaker implantation. He was discharged and is doing well at 24-month follow-up.

**Fig. 2:**
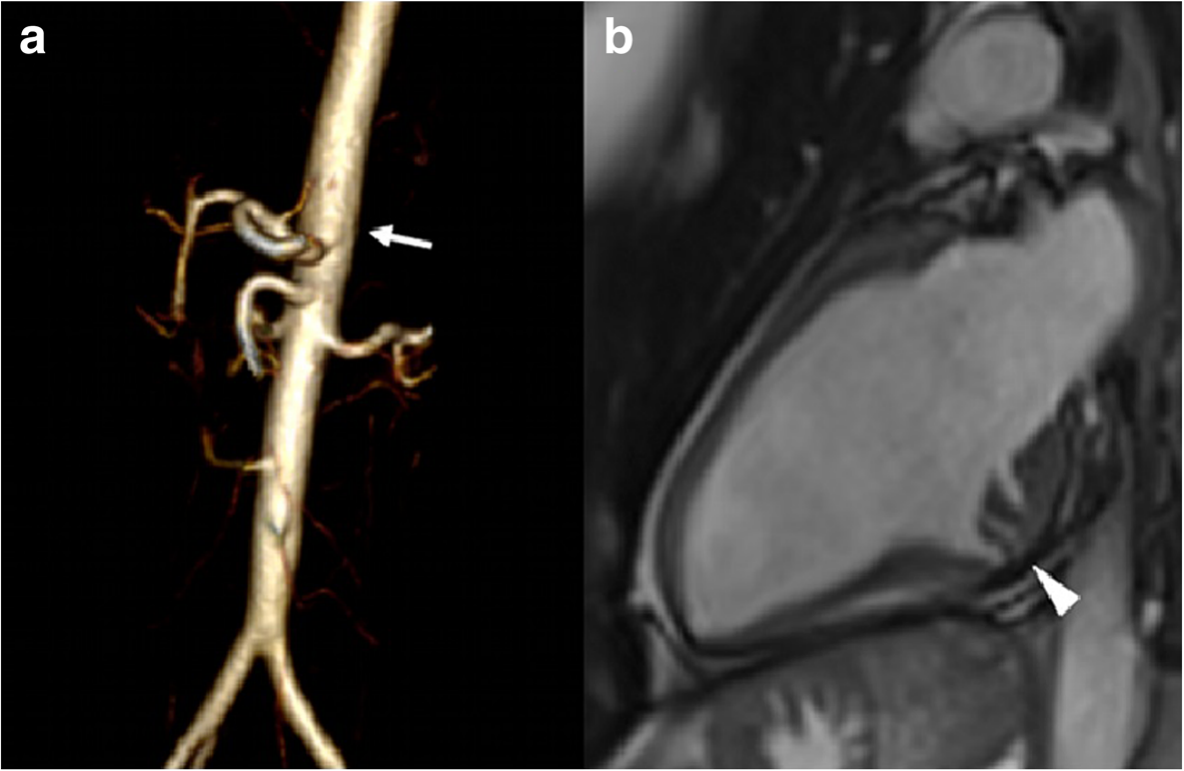
Magnetic resonance (MR) images of the child’s mother carrying low grade *TGFBR1* mosaicism. **Panel a**: volume rendering reconstruction of gadolinium-enhanced MR angiography showing ectasia of the proximal abdominal aorta (arrow). **Panel b**: vertical long-axis cine image of the left ventricle in the diastolic phase showing multiple myocardial crypts in the infero-basal segment (arrowhead)

In order to exclude subtle vascular abnormalities in the mother, a head to pelvis MRA was performed, which confirmed vascular asymmetry in the head and neck regions with predominance of the right vertebral and left carotid arteries without significant signs of arterial tortuosity. Volume rendering reconstruction of gadolinium-enhanced MRA showed mild tortuosity at the aortic arch and mild dilatation of the proximal abdominal aorta (20 × 20 cm) (Fig. 2a). Interestingly, multiple deep myocardial crypts in the inferior basal segment of the left ventricle were documented (Fig. 2b) along with anomalous distribution of the mitral papillary muscle without signs of regurgitation. Left ventricular function was completely normal.

Written informed consent was obtained from the patient’s parents for publication of this case report and all accompanying data.

We report this family because patients with LDS are commonly at high risk for acute vascular events (hemorrhage, dissection and sudden death) and less frequently known to be at risk for myocardial involvement and ventricular dysfunction (primary myocardial dysfunction due to heart muscle abnormalities and secondary myocardial dysfunction due to valvular and coronary vessel disease). No specific genotype-phenotype correlations have been observed in LDS. Somatic mosaicism is a rare finding but carrier patients are at risk of developing variable clinical manifestations, and its potential clinical implications for the cardiovascular system might be underestimated. In this paper, the novel findings of mosaicism and myocardial involvement in LDS are discussed.

We describe a maternally inherited *TGFBR1* mutation in mosaicism. The mother showed recurrent buccal artery hemorrhage that was managed surgically. She also experienced severe reduction in visual acuity secondary to unexplained vitreous detachment. Cardiovascular MRA showed generalized cerebrovascular asymmetry, mild dilatation of the proximal abdominal aorta, and multiple deep myocardial crypts in the inferior basal segment of the left ventricle, with a dysplastic aspect of the mitral papillary muscle architecture (Fig. 2).

## Discussion

The co-occurrence of two mendelian conditions in the same individual is increasingly described in literature mainly to advances in molecular methods that can cover multiple molecular pathways in only one experiment. In fact, in a recent review describing up to 4.9% of multiple molecular diagnoses of cases in which whole-exome sequencing was informative. These results described that structured clinical ontologies might be used to determine the degree of overlap between two mendelian diseases in the same individual; the diseases can be distinct (involving different systems or organs) or overlapping (interact at a certain way) [3]. Regarding our patient, the two mendelian conditions, namely LDS and OCA are distinct with different and well delineated features from each others. Paternal age at birth was 50 years, where initially advanced paternal age might be thought as a potential cause for predisposition to mendelian disorders but none of the molecular results of our patient coincide with this fact. In addition, our patient showed a large left cererbal arachnoid cystic formation in the frontal insular region (diameters 87 × 67 × 32 mm) with significant pressure effect on the surrounding cortex and mild hemispheric shift from the midline. Intracranial arachnoid cyst can be distinguished from other lesions containing cerebro-spinal fluid such as porencephalic cyst, first of all because arachnoid cyst location is usually peripheral within the subarachnoid space while porencephalic cyst is often central or lobar and surrounded by gliotic brain tissue. Arachnoid cyst is usually asymptomatic while porencephalic cyst often follows a history of trauma or stroke.

Molecular diagnosis of rare diseases is crucial for assessing actionable practical management, the recurrence risk and for addressing future prenatal diagnosis. In this specific report, the homozygous causative variant detected in *TYR* explains OCA clinical presentation and, as for all recessive diseases, the heterozygous carrier parents presented a 25% recurrence risk. *TGFBR1* identified variant, consisting with LDS features observed in the child, was inherited from the misdiagnosed mildly affected mother, presenting most probably the variant in both germline and somatic mosaicism. The recurrence risk of similar condition in future pregnancies strictly depends on the percentage of germline mutated cells percentage in gonads and hence cannot be established a priori.

Mosaicism is characterized by the presence of genetically distinct cell populations within the same individual secondary to postzygotic mutational events. The percentage, distribution in different organs, degree of tissue involvement are all related to the onset of this issue in relation to the stage of onset during embryogenesis. In fact if it occurs prior to differentiation of the germ line the soma maybe involved resulting in somatic-gonadal mosaicism [4].

Regarding somatic mosaicism, as emerged from the literature review, previous descriptions of LDS parents mosaicism is referred to paternally inherited mutations in *TGFBR2* but never in *TGFBR1* (Table 1). The first description reported *TGFBR2* somatic mosaicism (c.1609C > G, *p.Arg537Gly*) in a father without craniofacial manifestations, requiring aortic root replacement [5]. The second report presented the case of a father with *TGFBR2* somatic missense mutation in mosaicism c.1336G > A *(p.Asp446Asn)* [6]. His son showed the classic picture of LDS [7]. Mutation analysis of paternal DNA extracted from blood leukocytes, buccal cells, hair root cells, and nails indicated 52, 25, 0, and 35% of cells harbored the mutation, respectively. The father’s physical examination confirmed micrognathia and bifid uvula but limited vascular screening denied aortic changes. The third report documented a *TGFBR2* deletion identified by array CGH in a patient with microcephaly, mild dysmorphic features, and global developmental delay without any other features of LDS. The authors suggested that *TGFBR2* haploinsufficiency might be insufficient to cause the aortic phenotype of LDS but may result in craniofacial and skeletal abnormalities [8]. However, it is really difficult to predict outcome of toddlers since progressive vascular changes are a main characteristic of LDS and disease expression is often age-related. FISH analysis performed in the patient’s parents was negative but PCR analysis of DNA samples revealed somatic paternal mosaicism. Paternal phenotype was unspecified.

**Table 1:** Previously described patients with *TGFBR1* and *TGFBR2* mosaicism. Three patients showed paternally inherited *TGFBR2* in mosaicism, whereas our patient was a maternally inherited *TGFBR1* mutation carrier

	*TGFBR2*			*TGFBR1*
	Loeys et al., 2006 [5]	Watanabe et al., 2008 [6]	Campbell et al., 2011 [8]	Present report Past reported mutation by Mátyás et al., 2006 [2]
Molecular analysis	*R537G*	*D446N*	3p24.1 microdeletion	*R487Q*
Age at observation	unspecified	5 years	20 months	7 years and 4 months
Proband cardiovascular abnormalities	+(unspecified)	+	–	+
Arterial tortuosity	?	+	–	+, mainly intracranial
Arterial aneurysm/ dilatation	?	+, aortic root	–	+, severe aortic root
Arterial hypoplasia	?	+, bil. Subclavian and vertebral arteries	–	–
Congenital heart defects	?	+, VSD and BAV	–	–
Proband craniofacial abnormalities	+ (unspecified)		+	+ mild
Hypertelorism		+	unspecified	–
enophthalmos		+	+	–
cleft palate/bifid uvula		+	unspecified	+bifid uvula
malar hypoplasia		+	unspecified	–
Other dysmorphic features			Microcephaly, overfolded superior ear helices	Macrosomia. Oculocutaneous albinism
developmental delay		unspecified	+	–
Proband musculoskeletal abnormalities	unspecified	+	unspecified	+
pes planus		+		+
thumb camptodactyly		+		–
joint laxity		+		+
Others				Bilateral inguinal hernia
Parent with mosaicism	Father	Father	Father	Mother
Analysed tissue and percentage	Blood (unspecified %)	Blood (52%), buccal (25%), hair root (0%), nails (35%)	Blood lymphocytes and lymphoblasts (unspecified %)	Blood (18%), buccal (18%), hair root (10%)
Parent cardiovascular abnormalities	+	-ve 1st level vascular investigations	unspecified	+
Arterial abnormalities	aortic root replacement at age 45 yr)	–		Mild aortic dilatation, surgically repaired buccal artery for repeated hemorrhage
Others		–		myocardial inferobasal crypts and abnormal mitral and myocardial fibers distribution
Parent craniofacial abnormalities	–	+	–	–
Bifid uvula	–	+	–	–
Jaw abnormalities	–	+	–	–

The previous few but significant examples reporting parents with somatic mosaic condition in LDS express wide variability in expression of the disease. It varies from “apparent” incomplete penetrance, milder phenotype, unusually described features down to major cardiac involvement. It is well known that the access to specifically involved tissues is unfeasible in the majority of cases. However, in clinical practice there is the trend to investigate tissues with less invasiveness such as in our case and some of the above mentioned conditions for testing hair bulb and saliva. If germline mosaicism is involved the transmission of severer condition can be expected and the recurrence risk will be higher but it is difficult to estimate in terms of numerical values.

Myocardial involvement in LDS is a rare finding. Our search criteria (PubMed) included the association of LDS with heart failure, cardiomyopathy, and myocardial changes. Based on search results, LDS is well recognized to predispose patients to acute vascular events but less is known on its association with heart muscle disease and dysfunction (Additional file 1: Table S1). In LDS, macrovascular disease is secondary to mutations of transforming growth factor receptor. The contribution of microvascular dysplasia in LDS has previously been hypothesized [9], given that microvascular dysplasia is expected to impair myocardial blood flow, likely resulting in myocardial dysfunction. Transforming growth factor-beta plays a major role in myocardial hypertrophy and vascular remodeling in adults [10, 11]. Moreover, it is essential for the formation of endocardial cushions, the coronary vasculature and the ventricular myocardium during embryogenesis. The observation of multiple deep myocardial clefts in the proband’s mother is hypothesized to be a minimal manifestation of subtle myocardial disease. This finding is reported in 6% of healthy volunteers, whereas it is more prevalent in patients with hypertrophic cardiomyopathy (16%), myocarditis (15%), and hypertension (14%). In particular, myocardial clefts are frequently reported in hypertrophic cardiomyopathy mutation carriers without left ventricular hypertrophy [12, 13]. It is likely that myocardial crypts are due to failure to resorb the trabeculated part of the ventricular wall during the normal embryological process, most often located in the basal half of the left ventricular chamber [14]. However, at present, no long-term data are available on the clinical significance of myocardial clefts in normal or hypertrophic cardiomyopathy subjects. Several studies suggest that they might be part of an early phenotypic hypertrophic cardiomyopathy marker [13].

## Conclusions

Additional case descriptions are needed to draw any conclusions regarding the phenotypic spectrum of patients with *TGFBR1/2* mutations in mosaicism. Reporting on subtle vascular symptoms, as in our case, can help identifying mild forms. Further studies with large cohorts are warranted to correlate the phenotypic spectrum of heart muscle abnormalities with LDS. In the reported case, the long-term follow-up of the child’s mother included primary and secondary vascular imaging to exclude as early as possible any vascular and myocardial impairment. However, at present and with the methods used, we are unable to differentiate hemorrhagic events in the mother of index patient reflecting the consequences of a TGFBR1-related vascular malformation or resulting from another cause. Careful evaluation of myocardial imaging combined with assessment of the vascular territory is recommended for the early detection of progressive cardiovascular abnormalities in LDS patients.

## Additional file

Additional file 1: **Table S1.** Literature review of LDS patients showing systolic dysfunction and/or myocardial changes. (DOCX 15 kb)

## Abbreviations

CMA: Chromosomal microarray analysis
LDS: Loeys-Dietz syndrome
MRA: Magnetic resonance angiography
NGS: Next generation sequencing
OCA: Oculocutaneous albinism
