# Supplementary material for: First evidence of maternally inherited mosaicism in TGFBR1 and subtle primary myocardial changes in Loeys-Dietz syndrome: a case report
Source: BMC Med Genet. 2018 Sep 15;19:170. doi: 10.1186/s12881-018-0661-2 (PMC6139163; doi:10.1186/s12881-018-0661-2)
Supplement: Supplementary file 1 — Table S1. Literature review of LDS patients showing systolic dysfunction and/or myocardial changes. (DOCX 15 kb) [file 12881_2018_661_MOESM1_ESM.docx]

| **Report** | **Gender** | **Age** | **LDS** | **Genetic finding** | **Extracardiac features** | **Vascular features** | **HF/ myocardial features** | **Comments** |
| --- | --- | --- | --- | --- | --- | --- | --- | --- |
| **O’Reardon et al., 2008^1^** | F | 45y | Yes | Unspecified | negative | negative | Takotsubo CMP (stressed induced CMP) | Major depression |
| **Eckmann et al., 2009** | M | 44y | Yes | TGFBR1: 1303G>C | wide uvula, high arched palate,ankyloglossia, retrognathia, pinched nasal bridge, mild joint laxity, and pectus carinatum | Acute thoracoabdominal aortic dissection | At 1month postop: AHF, LVEF 25% | Heart transplanted specimen: interstitial and replacement fibrosis ofthe LV myocardium and dysplastic medium-to-small coronary arteries |
| **Yamana et al., 2017** | M | 14y | Yes | Unspecified | bilateral club foot | type A aortic dissection, moderate aortic regurgitation and aortic root aneurysm | At 1month postop: AHF, LVEF 30% | Long term improvement after staged surgical repair and antiHF medical treatment |
| **Present Report** | F | 50y | Yes | TGFBR1: c.1460G>A | pinched nasal bridge | Mild aortic dilatation, surgically repaired buccal artery for repeated hemorrhage | myocardial inferobasal crypts and anomalous distribution of papillary muscles |  |

**Reference of Table S1**

1. O’Reardon JP, Lott JP, Akhtar UW, Cristancho P, Weiss D, Jones N. Acute coronary syndrome (Takotsubo cardiomyopathy) following electroconvulsive therapy in the absence of significant coronary artery disease: case report and review of the literature. *J ECT* 2008; 24:277-280.
